# Machine learning-based unified models for predicting drug clearance from pharmacokinetic animal and study design variables

**DOI:** 10.1371/journal.pone.0346432

**Published:** 2026-05-06

**Authors:** Remya Ampadi Ramachandran, Lisa A. Tell, Melissa A. Mercer, Xuan Xu, Nuwan Indika Millagaha Gedara, Maaike Ottoline Clapham, Zhoumeng Lin, Jim E. Riviere, Majid Jaberi-Douraki

**Affiliations:** 1 1DATA Consortium, www.1DATA.life, Kansas State University Olathe, Olathe, Kansas, United States of America; 2 Food Animal Residue Avoidance and Databank Program (FARAD), Kansas State University Olathe, Olathe, Kansas, United States of America; 3 Department of Mathematics, Kansas State University, Manhattan, Kansas, United States of America; 4 Department of Medicine and Epidemiology, FARAD, School of Veterinary Medicine, University of California-Davis, Davis, California, United States of America; 5 Department of Statistics, Kansas State University, Manhattan, United States of America; 6 College of Business, Loyola University, New Orleans, Louisiana, United States of America; 7 Department of Environmental and Global Health, College of Public Health and Health Professions, University of Florida, Gainesville, FL, United States of America; 8 Center for Environmental and Human Toxicology, University of Florida, Gainesville, FL, United States of America; 9 Department of Population Health and Pathobiology, FARAD, College of Veterinary Medicine, North Carolina State University, Raleigh, North Carolina, United States of America; Universidad El Bosque, COLOMBIA

## Abstract

Clearance (CL) is a primary pharmacokinetic (PK) parameter crucial to determine how quickly a drug is eliminated from the body, which guides the appropriate dosing interval to maintain a consistent concentration in blood. Given the importance of CL, this study aimed to use machine learning (ML) techniques to predict CL values by identifying patterns and relationships within an extracted dataset of PK variables from published articles. Variables evaluated in the extracted dataset included drug, dose, animal species, and route of administration. Nine distinct ML models were then applied to analyze the CL data, incorporating both imbalanced and balanced data generated through resampling methods. Since the CL data used in this study is a collection of all CL values (true CL and CL/F) extracted from scientific articles, the collected CL variable for both IV and non-IV administration routes are referred to as hybrid ML CL. To analyze the effect of ML models in predicting the CL values, we used the hybrid ML CL dataset for six different subsets of data including one solely from the intravenous route of administration. Linear regression, multi-layer perceptron, and random forest models consistently had the highest efficiency in predicting CL values, with an R^2^ score > 0.87. However, R^2^ increased to > 0.95 when analyzing only ungulates or small ruminants, and > 0.92 for the companion animal group. This study has the potential to help researchers employ computational, mathematical, and ML models to predict and estimate CL values and changes in CL values based on variables. This study focuses on evaluating the feasibility of predicting drug CL in situations where direct CL data are not available. Rather than addressing drug development processes, the research examines whether study design variables can serve as input parameters for a proposed cross-species extrapolation tool aimed specifically at predicting existing drug CL values.

## Introduction

Veterinary drugs play a vital role in maintaining the health of both companion and agricultural animals. Since veterinary drugs are evaluated for safety and efficacy in the target species at the earliest stages of drug development, early and successful dose characterization is critical to the veterinary drug development pipeline [1]. While there are many similarities between human and veterinary pharmaceutical development, product developers face unique challenges when bringing veterinary drugs to market. Factors such as disease, species, age, animal breed/genetics, and physiologic state (including pregnancy, lactation, or disease) can significantly impact a drug’s pharmacokinetics (PK) [2–7]. Therefore, the development of in silico PK modeling methods may be particularly advantageous to improve the efficiency of assessing target dosing strategies during preclinical drug development.

The two most critical PK parameters for dose characterization are the Volume of Distribution (Vd) and Clearance (CL). Vd is a proportionality constant equal to the amount of drug in the body divided by the plasma concentration at a given time and is primarily used for an appropriate loading dose. Since Vd is primarily determined by the drug’s physical properties, including lipophilicity, water solubility, charge, and extent of protein binding, Vd is relatively straightforward to successfully model via a variety of in silico approaches [8]. Meanwhile, CL is the PK parameter that measures the ability of the body to eliminate a drug per unit of time. As such, CL is the relevant parameter to calculating the dose and dosing interval required to maintain the steady-state plasma concentration of a drug. CL is also used to model the terminal half-life of a drug and to predict potential drug-drug interactions.

In contrast to Vd, CL has proven to be a far more challenging PK parameter to model. Total body clearance (CL_T_) is a summation of all the individual organ CLs, including liver and kidney. The reason modeling CL is challenging is that CL_T_ is influenced by both drug properties and animal physiology. Consequently, accurately modeling CL_T_ requires accounting for the complex interactions between drug metabolism and organ-specific physiology [9]. CL_T_ can be affected by factors such as species, genetics, age, physiologic status, and disease—all of which must be considered when developing a dosing regimen [3,4,10]. In vivo CL_T_ is best determined following intravenous (IV) administration, as this method bypasses the variability introduced by drug absorption (as reflected by drug bioavailability [F]). However, when in vivo studies are conducted using extravascular routes of administration, bioavailability (F) is not directly measured, and clearance is often reported as CL/F, which further increases the challenge of modeling CL.

Approaches that predict individual organ CL, such as hepatic CL, based on in vitro techniques have been extensively explored in small molecule development for humans [11] and in a limited fashion to predict hepatic CL in veterinary species [12]. However, these in vitro techniques can only predict hepatic CL, not the CL_T_ used for dose characterization. Therefore, CL_T_ has most commonly been predicted using in silico methods, be that allometric scaling [13,14], in vivo–in vitro extrapolation (IVIVE) [15], or physiologically based pharmacokinetic (PBPK) modeling [16]. These methods have variable accuracy when compared to in vivo data and are based on individual studies or small pools of data to predict PK parameters, which may not fully reflect the variability of the target population.

Artificial intelligence (AI) and Machine Learning (ML) have been explored as a method for predicting CL_T_ in various species including preclinical species [17–19]. ML methods incorporate large pools of data in a variety of ages, weights, and physiologic states to predict parameters based on broad population-based data. These methods have been applied to predict plasma half-lives and CL_T_ of drugs in veterinary species using molecular descriptors [20,21], but have not yet been used to explore the ability to predict CL_T_ in veterinary species using the pharmacokinetic study design parameters for individual drugs. The proposed AI/ML model focuses on data harvesting from scientific literature and prediction of true CL which includes the IV-only CL dataset as well the prediction of hybrid ML CL where the dataset includes both true CL and CL/F values, based on pharmacokinetic animal study design variables. This model stands apart because it primarily chooses the PK study design variables as the predictor or independent variables, unlike other mathematical and ML PK models that rely on molecular descriptors and physicochemical properties [21,22].

Although robust pharmacokinetic data is fundamental for understanding drug disposition, CL values are often missing or difficult to obtain due to resource limitations, ethical constraints, or the absence of comprehensive in vivo studies. To address these practical barriers, the present study investigates whether study design variables alone can be leveraged to reliably predict drug CL values when empirical data are missing. By focusing the analysis on existing drugs, the proposed cross-species extrapolation tool is used to explore the potential of study design variables as inputs in generating accurate CL predictions. This study aimed to evaluate the efficacy of nine distinct ML models to estimate total body CL in a variety of veterinary species.

## Methodology

### Data resources

Similar to other ML methods, collecting data is a vital part of developing an automated drug CL_T_ prediction model. Due to the limited accessibility of the preexisting CL_T_ database suitable for ML research, we relied on scientific literature to create our dataset [22,23]. Our automated Web Crawler for PK was utilized to download XML versions of scientific articles [24]. The database we utilized for this study comprised scientific publications in XML format, obtained mainly from the Scopus, Springer, and Crossref TDM API providers. These publications primarily cover the following Anatomical Therapeutic Chemical (ATC) drug classes: QD01–QD11 (Dermatologicals), QH01-QH05 (Systemic Hormonal Preparations Excluding Sex Hormones and Insulin), QJ01–QJ05 (Antiinfectives for Systemic Use), and QP51–QP54 (Antiparasitic Products, Insecticides, and Repellents). Based on the ATC classification, some of these drugs may belong to other drug classes including QMs (NSAIDs – Anti-inflammatory and antirheumatic products). The ATCvet code used here is formed by adding the letter Q to the standard ATC code [25,26]. In both the ATC and the ATCvet systems, preparations are categorized based on their intended therapeutic use (Level 1: broad anatomical groups QA – QV), preparations are further subdivided into therapeutic main groups (Level 2: QA01, QA02, etc.), categorized into chemical, therapeutic, or pharmacological subgroups (Levels 3: QA02A, QA02B, etc., and Level 4: QA02AA, QA02AB, etc.), and finally the chemical substance classification except for QI Immunological (Level 5: QA02AA01).

### Data collection

As part of the automated data extraction procedure, the XML versions of scientific articles were given as input to table the data extraction module to identify their table format and extract corresponding PK data [24,27–29]. Likewise, in this study, the automated modules implemented using the Python programming language (version 3.8) [30] generates an output dataset focused on developing drug clearance prediction model. Table 1 indicates a sample output generated in.csv file format from the table data extraction and curation module for the DOI 10.1016/j.thromres.2015.07.019  [**31**].

**Table 1:** A sample dataset generated from the table data extraction and curation module for the Table 5 of DOI 10.1016/j.thromres.2015.07.019 [31], with DOI, table number, title of the article, drug name, dosage, route of administration, animals, number of animals, and clearance values in the columns respectively.

DOI	Table No:	Title	Drug	Dose	Route	Animal	Animal No:	CL
10.1016/j.thromres.2015.07.019	5	Evaluation of the toxicology and pharmacokinetics of recombinant factor VIII Fc fusion protein in animals	rFVIIIFc	3000 IU/kg	intravenous	Monkey	2	16.3 mL/h/kg
10.1016/j.thromres.2015.07.019	5	Evaluation of the toxicology and pharmacokinetics of recombinant factor VIII Fc fusion protein in animals	rFVIIIFc	10000 IU/kg	intravenous	Monkey	2	38.6 mL/h/kg
10.1016/j.thromres.2015.07.019	5	Evaluation of the toxicology and pharmacokinetics of recombinant factor VIII Fc fusion protein in animals	rFVIIIFc	20000 IU/kg	intravenous	Monkey	2	42.0 mL/h/kg

Manual extractions were also conducted to verify and validate the precision of the automatic extraction process and to fill in any missing data. In this way, data present in tables of extracted scientific articles were curated both manually [32] and by using an automated extraction approach implemented in Python modules. Finally, for this study, we have limited our data collection and curation to the CL data for every drug, route of administration, and animal, that were extracted from the published records [24] and could be accessed through the rule-based tabular data extraction module for the XML version created in Python [30]. The rule-based tabular data extraction method is similar to the named entity recognition (NER) model that addresses the challenges in accurately extracting the PK parameters from scientific literature [33]. Additionally, a database of pharmacokinetic data manually extracted from scientific articles by the Food Animal Residue Avoidance and Depletion (FARAD) program was included [32]. Therefore, due to the variety of different routes of administration, the extracted CL data used in this study included both CL and apparent CL (CL/F). As such, the CL data parameter in this study was termed a hybrid route-independent machine learning CL (hybrid ML CL_T_), which is a combination of CL and CL/F values from both extravascular and intravascular drug administrations.

### Data compliance

The dataset used in this study was both manually and automatically curated from scientific publications indexed mainly in PubMed and Scopus databases. Relevant articles were identified using keyword-based searches and the data including clearance, drug, dosage, route of information, and animal/species were systematically extracted. Because all the data were obtained from open access or subscription based publications, used in compliance with the terms and conditions of the respective bibliographic sources, no prior approval was required. The data collected, through the two methodologies employed in this study, namely the automated customizable web crawler [24] and the manual collection [32], together with the data analyzed and shared, adhered to the terms and conditions of the data sources, along with relevant ethical and legal standards.

### Data pre-processing

To ensure data quality, data cleansing was incorporated in the initial phase of model development which helped to merge manually and automatically collected datasets; remove unwanted observations, manage unwanted outliers, handle or rebuild missing data, de-duplicate, unit standardization, and resulted in a cleaned dataset. As part of generating a valid dataset for this study, we used experts’ opinion in this data preparation phase to determine the range of clearance values, as mentioned in the Data Sampling section below, used in this study from the dataset curated. Data preprocessing is the term coined to represent such data manipulation or discarding data to generate high-quality datasets for implementing high-performance ML models [34].

Drug CL can be expressed as (volume/time) and is commonly normalized to body weight for veterinary species. As such, the CL values published in the articles were expressed in a variety of units, including L/h/kg, mL/min/kg, μL/min/mg, mL/h/kg, and similar variations. To address the variety of different units for CL, we also converted the collected CL data into a unit standardized format (mL/min/kg). After cleaning the dataset, it was divided into two datasets for training and testing the models. The performance, reliability, and generalizability of the machine learning model were confirmed by using the 5-fold cross-validation scheme. Here, instead of performing a single train-test split, the training dataset was randomly divided into five equal-sized subsets or folds. The model was then trained on four of the folds and evaluated on the remaining fold. This process was repeated five times, with each fold serving as the test set exactly once [34].

### Data sampling

**Fig 1:**
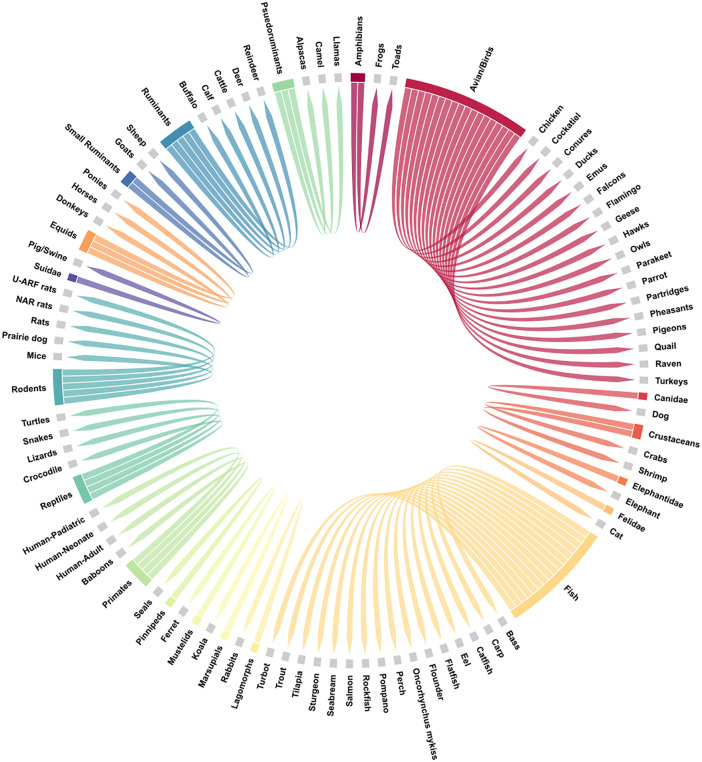
Circular dendrogram representing various animal grouping strategies that can be considered in the PK parameter prediction models. Some of these groups were considered as independent candidates for clearance prediction in this study.

The data obtained from the pre-processing module often had an imbalance in nature relative to class distributions (drugs or animal groups shown in Fig 1) of unequal size, yet most ML algorithms deliver optimal outcomes for balanced class distributions [34,35]. For example, there are not equal numbers of papers published across species (e.g., there are far more PK studies performed in cattle than camelids), and certain routes of administration are more commonly studied than others, based on the feasibility of administration of certain species. Duplicating or creating synthetic datasets for minority classes (*oversampling),* dropping data from majority classes (*undersampling)*, or using a combination of both approaches (*simultaneous sampling)* have been utilized to analyze imbalanced datasets before fitting the ML models. Python imbalanced-learn library which was imported as *imblearn* has been employed with RandomUnderSampler and RandomOverSampler for undersampling and oversampling techniques, respectively. *Imblearn Pipeline* has been used to implement the resampling technique from the selected methods [36,37]. In this manner, along with the implementation of binning strategies to handle continuous variables or targets, flexible response-stratified sampling strategies can be applied within regression framework. This approach addressed imbalance issues in the datasets while preserving the continuous nature of the target clearance values, ensuring the ML model’s ability to perform accurate prediction.

In this way, the literature-derived clearance data were curated and harmonized by converting reported values to a consistent unit (mL/min/kg), incorporating key study variables (drug, dose, route of administration, and species), and applying predefined aggregation and outlier screening procedures, with domain-informed review used to resolve ambiguous or sparsely reported entries. Because the resulting dataset exhibited an uneven distribution across clearance ranges, resampling strategies were applied within the training pipeline to mitigate data imbalance during model development.

### Dataset

**Fig 2:**
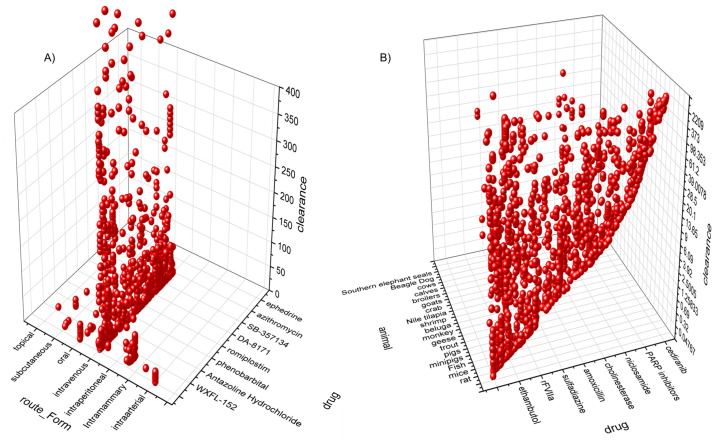
3D scatter plot representing the dataset (raw/imbalanced) used to develop the clearance/clearance/F(bioavailability) prediction model. This plot contains a representative dataset used in the model development (A) clearance data distribution corresponding to drug and route of administration, (B) clearance data distribution corresponding to drug and animal.

Modeling predictions of true CL or CL/F in this study was achieved using datasets that included drug, dose, route of administration, and species, which were extracted from peer-reviewed published pharmacokinetic studies using live animals or *in vitro* studies. A total of 483 drugs, 18 routes of administration, and 52 species having different combinations of CL_T_ values were curated. Fig 1 and 2 (https://1data.life/pages/publication/ML_Predicting_Drug_Clearance_PK/Cl_Paper_Fig1.svg) present a general overview of the datasets extracted. S1 File (https://1data.life/pages/publication/ML_Predicting_Drug_Clearance_PK/Cl_Paper_Supplment_S1_.html) includes different data clusters with species and drugs gathered from research literature in their raw state. The pre-processing module refined these curated datasets by consolidating drug names, routes of administration, and species to create a clean, consistent dataset. Fig 2 and Table 2 describe the data after eliminating the multiple occurrences of various data combinations with their drug and CL_T_ values. In the data preprocessing phase, we placed high importance on removing any records that had missing information in any of the columns, including drug, dose, route, species, and clearance. It also handled grouping or assigning the same name to different representations of the same route forms, such as ‘IV’, intravenously’, and ‘intravenous’, as shown in Table 2. Additionally, the *in vitro* routes reported in Table 2 were automatically extracted by the table data curation module [24,27], but they were not all used for the ML model development, as they do not reflect real routes of administration, but rather an experimental scenario.

**Table 2:** An example of the routes of administration curated from research literature and the associated short forms (abbreviations) assigned for building ML models. Note: not all listed routes of administration were included in ML model development.

Route	Abbreviation	Route	Abbreviation
Capsule*	PO	Intraportal(ly) or IPO	IPO
Intramammary or IMM	IMM	Intravenous(ly)	IV
Inhalation or INH	INH	IV bolus	IVB
Intraarterial(ly) or IA	IA	Oral(ly) or PO	PO
Intragastric(ally) or IG	IG	Parenteral(ly) or PAR	PAR
Intramuscular(ly) or IM	IM	Subcutaneous(ly) or SC	SC
Intraperitoneal(ly) or IP	IP	Topical(ly) or TOP	TOP

* depicts the route form discussed in some article discussions, but it is treated as oral (PO) for the ML models in this study.

### Feature importance score

In ML model development, a crucial step involves calculating the scores of all predictor variables in a model in order to assess their importance in the decision-making or prediction process. In the proposed CL_T_ prediction model, the feature importance of a forest of trees has been considered [38]. Here, the fitted attribute feature importances (feature_importances_) were determined by calculating the average and standard deviation of the decrease in impurity for each tree in the model. By using this approach, we have the flexibility to perform feature importance calculations either by *mean decrease in impurity* or *feature permutation*. Relying on feature permutation to measure feature importance can eliminate bias towards high-cardinality features, which is a drawback of using impurity-based feature importance calculation [34]. This research additionally assessed the significance of features using SHAP (Shapley Additive exPlanations) analysis [39]. The RF ranks feature contributions globally, supporting features with continuous values due to its regular and effective data splitting across all trees, and it calculates the features’ overall contributions to accuracy or impurity reduction. On the other hand, SHAP values apply cooperative game theory to assign a fair, instance-specific credit of each feature’s contribution to predictions, which may be aggregated for overarching insights. Due to these reasons, RF feature importance scores and SHAP values may show complementary insights into model interpretability resulting in potentially different feature contributions.

### Performance measures

The efficiency of an ML model in making predictions is measured by its quality assessment. The assessment is made using various model evaluation tools including cross-validation scores or metric functions like scoring parameters. The cross-validation score relies on an internal scoring strategy, involving splitting the dataset into k consecutive folds, with each fold being allocated to the validation phase and the others to the training set. This helps to prevent problems of overfitting. In the case of predicting continuous target values, regression models are preferred over classification models. Since our proposed study was focused on predicting drug CL_T_ values, which is a continuous variable, we opted to use ML regression models. The performance of these models was evaluated using *r*^*2*^ *scores* as the primary performance metrics. The *r*^*2*^ *scores* evaluated indicate the model’s capability of ‘how perfectly the model is trained for the given dataset’. An ideal model is the one with a r^2^ score of 1 or close to 1 which in turn confirms the efficiency of the model that we implemented for predicting the CL_T_ values. It can be calculated using Equation (1) [34,40,41].


$$r^{\text{2}}=\text{1}-\frac{{\sum (y_{i}-\hat{y})}^{\text{2}}}{{\sum (y_{i}-\overset{―}{y})}^{\text{2}}}$$ (1)

where $\hat{y}$, and $\overset{―}{y}$ are the predicted, and actual values of y, respectively.

The *explained variance score* (EVS) represents the amount of error dispersion present in a particular dataset as calculated using Equation (2). Similar to the r^2^ score, values closer to 1 are preferable.


$$explainedvariancescore\left(y,\hat{y}\right)=\text{1}-\frac{Var\left(y_{i}-\hat{y}\right)}{Var(y)}$$ (2)

where $Var(y_{i}-\hat{y})$ is the variance of prediction errors, and Var(y) is the variance of actual values.

Root mean square error (RMSE), which is an extension of mean square error (MSE), is recognized as the predominant error measure of precision in regression, quantifying performance in the same units as the predicted values. RMSE values can be computed using equation (3), with a score of 0 being deemed ideal:


$$RMSE=\sqrt{\frac{\text{1}}{N}\sum \overset{N}{\underset{i=\text{1}}{\mathrm{\backslash nolimits}}}{\left(y_{i}-\hat{y}\right)}^{\text{2}}}$$ (3)

The mean absolute error (MAE) metric helps us understand how different our predictions are from the actual values in the dataset. It assists in assessing the accuracy of the model by measuring the absolute differences between the predicted and real values (Equation (4)):


$$MAE=\frac{\sum_{i=\text{1}}^{n}abs({\overset{―}{y}}_{i}-\hat{y_{i}}))}{n}$$ (4)

where $\overset{\prime }{\underset{i}{\overset{―}{y}}}\text{s}$ are the actual values and $\hat{y_{i}}\text{'}\text{s}$ are the predicted values for i in range(n) with *n* being the length of actual values.

## Results

### Data sampler and machine learning models

**Fig 3:**
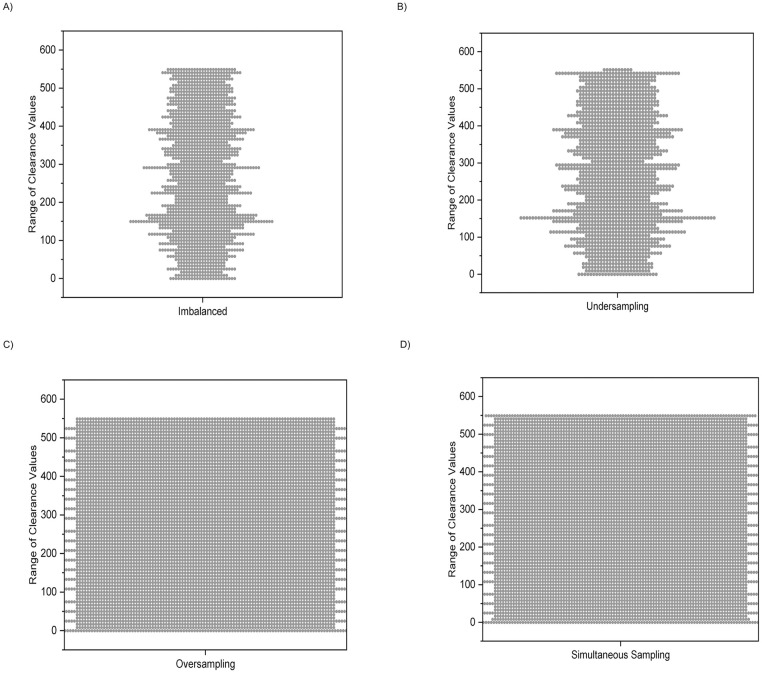
Distribution of datasets selected for the prediction models, (A) imbalanced, (B) undersampling, (C) oversampling, and (D) simultaneous resampling methods. Compared to Fig 3A, figures B, C, and D attain a well-balanced data distribution by modifying the frequency of data samples. This is accomplished by either decreasing or increasing the number of samples, using the Imbalanced-Learn Python Module.

A dataset comprising 4,788 records was extracted from the tables present in the XML files. There were 129 drugs with extractable data across 10 ungulate species (alpacas, buffalo, camels, cattle, donkeys, goats, horses, llamas, pigs, and sheep). There were 50 drugs with extractable data across 2 small ruminant species and 33 drugs with extractable data across 12 avian species. To evaluate the efficiency of ML models at predicting CL_T_ values, we separated certain groups from the overall data analysis for individual examination (cases), with a sample of the findings presented in this article. The PK data curated from the literature includes imbalanced observations of its data clusters such as species, route of administration, drug, and dosage, all of which affect the target variable clearance. Fig 3A confirms the imbalanced nature of the selected dataset.

As mentioned in the ‘Data Sampling’ section above, random resampling strategies such as random undersampling (Fig 3B), random oversampling (Fig 3C), and a combination of these strategies for generating simultaneously sampled (Fig 3D) datasets were applied [42]. Fig 3 is an illustration of the influence of various resampling strategies over the imbalanced dataset distribution. Similarly, a balanced distribution of classes was achieved by adjusting the frequency of data samples, either by reducing or increasing them. Undersampling (Fig 3B) reduces the number of samples in the original majority class, making it comparable to the original count of the minority class. However, because it works by removing samples rather than adding new ones, the resulting dataset may not appear as dramatically balanced as datasets created through oversampling (Fig 3C) or simultaneous (Fig 3D) sampling.

### Feature importance

The features in importance analysis refer to the predictor study design parameters for individual drugs such as route of administration, species, drug, or dose. Feature importance analysis conducted in this study shows the level of influence or percentage of the study design variables’ contribution to the corresponding target variable (CL). As per the feature importance score from the RF feature importance assessment, the CL_T_ value is primarily influenced by (in order of decreasing contribution) the drug, then the dosage, route of administration, and then the treated species. However, according to the RF feature importance score for the balanced dataset, the route of administration had a relatively lower impact on the CL_T_ value. This is not unexpected because the CL_T_ parameter being estimated by the ML model was the hybrid ML CL_T_, which includes CL/F which, through bioavailability, already accounts for the influence of the route of administration. Additionally, since all drug types were included, even though CL_T_ is typically dose-independent, dose-dependent CL could be observed for drugs with saturable metabolism.

**Fig 4:**
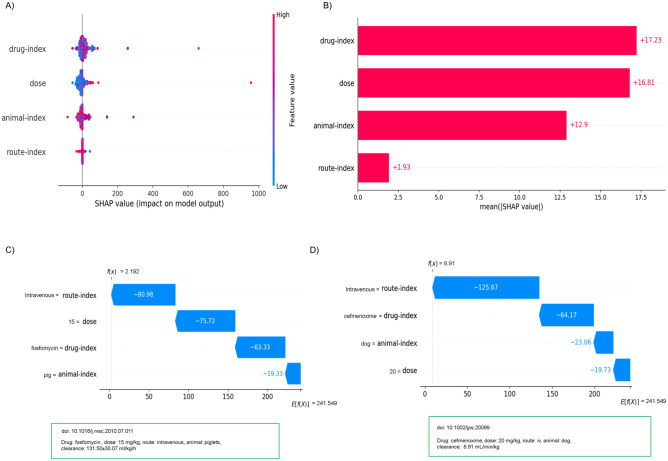
(A). SHAP summary plot, Figure (B): SHAP bar plot, (C) – (D): Representative SHAP waterfall plots depicting feature contributions to individual predictions. It shows, how each attribute contributes positively or negatively to predict the target values. E[f(X)] represents the base value which is the average model output from the SHAP implementation and functions as the reference point. Representative datasets with actual values, highlighted in green boxes, and its predictions represented as f(X). Note that, in this study the unit of clearance value is considered in mL/min/kg, and so the predicted value may find different from the one displayed in green boxes.

While Fig 4A–B depicts the contribution of each feature to the overall prediction of clearance values for the imbalanced dataset; Fig 4C–D depicts the feature contribution scores obtained for selected drug, dosage, animal, and administration route within the curated imbalanced datasets from the SHAP analysis. The figures also display the base value E[f(x)]=241.549 from SHAP implementation, and feature attributions that demonstrate the impact of each feature in modifying the prediction in relation to the base value. The base value indicates the average model output from SHAP implementation. In addition to the impact of specific feature contributions, these plots illustrate the closeness of the actual and predicted values for the specified dataset. SHAP contribution measures facilitate understanding of local interpretability, clarifying the reasoning behind the model’s prediction for a specific sample, in contrast to the global interpretability provided by RF feature importance measures.

**Fig 5:**
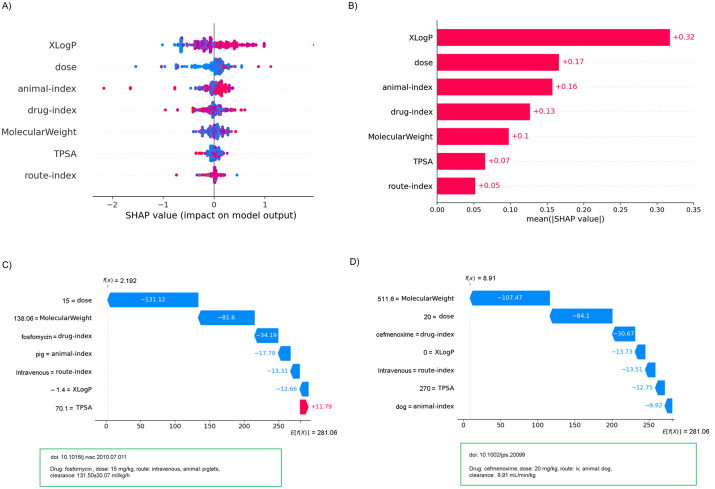
(A). SHAP summary plot, Figure (B): SHAP bar plot, (C) – (D): Representative SHAP waterfall plots depicting feature (study design variables and molecular descriptors) contributions to individual predictions. It shows how each attribute contributes positively or negatively to predicting the target values. E[f(X)] represents the base value which is the average model output from the SHAP implementation and functions as the reference point. Representative datasets with actual values, highlighted in green boxes, and their predictions represented as f(X). Note that, in this study the unit of clearance value is considered in mL/min/kg, and so the predicted value may find different from the one displayed in green boxes.

In the proposed model, we primarily examined how study design variables influenced the prediction of drug clearance values. To further evaluate the impact of molecular descriptors, we conducted a feasibility analysis comparing models based on study-design-only predictors, combined study-design + molecular descriptors, and molecular-descriptors-only. Fig 5A–B shows the results obtained from the prototype model with each feature’s contribution to the overall prediction of clearance for the imbalanced dataset, whereas Fig 5C–D provides the corresponding feature contribution score and prediction results obtained for the same combination of drug, dosage, animal, and administration route as shown in Fig 4C–D, now supplemented with Mw, LogP, TPSA values.

Compared to the model with study design variables alone, incorporating molecular descriptors modestly improved the performance scores to the higher R^2^ score of approximately 0.82 for the combined imbalanced dataset in both cross-validation and 70:30 training:test data split scenarios. In contrast, the molecular-descriptors-only model performed substantially worse, with an R^2^ score of approximately 0.54. These findings highlight both the feasibility of integrating molecular descriptors and the central importance of study design variables in predicting drug clearance for the combinations of predictors examined in this study.

### Prediction models

This paper presents ML prediction models for four subsets of the CL_T_ dataset using the PK study design variables as the predictor variables, resulting in a hybrid ML CL_T_ dataset for Cases 1–5. Cases 1–5 focused on animal categories and included all routes of administration (intravascular and extravascular): Case 1 – the total hybrid ML CL dataset curated from the literature for all animal categories, Case 2 – a subset of hybrid ML CL dataset focusing on ungulates, Case 3 – a subset of the ungulate hybrid ML CL dataset focusing solely on small ruminants, Case 4 – a subset of hybrid ML CL dataset focusing on companion animals, Case 5 – hybrid ML CL prediction models for different train:test data splitting options. Case 6 was a subset of the dataset (true CL) with only the intravenous (IV, IV bolus, or IV constant rate infusion) route of administration across all dosages and animal species. The performance of the models was evaluated under two different conditions: A) without implementing resampling techniques (imbalanced datasets), and B) with resampling techniques (balanced datasets) in predicting the drug CL or CL/F values.

Unlike other prediction models [43–47] that rely on molecular descriptors of drugs, structural characteristics, physiological data, or physicochemical properties, the ML prediction models discussed in this study use the study design variables of an individual drug including drug dosage, route of administration, and animal species as input variables to predict the hybrid ML CL_T_ (Case 1–5) and true CL_T_ (IV) (Case 6) rates of drugs. Unlike other ML models, the ML models presented in this manuscript focus on identifying patterns and relationships within the PK study design variable, including feature contributions and interactions, to make parameter estimations. We do not assess the clinical implications of the input variables nor attempt to make predictions for drugs not included in the analyzed datasets, thereby reducing the need for molecular descriptors of drug structure. The dataset is also used to assess what ML approach is optimal to estimate CL_T_ data. In the proposed ML prediction models (Case 1–5), all routes of administration, including intravenous (IV), are treated equally in determining the hybrid ML CL_T_ rates, while IV administration is considered the primary determinant of CL in pharmacological, clinical and structure-activity relationship models [43–47].

### Case 1 (all animal categories and route forms – hybrid ML CL_T_)

**Fig 6:**
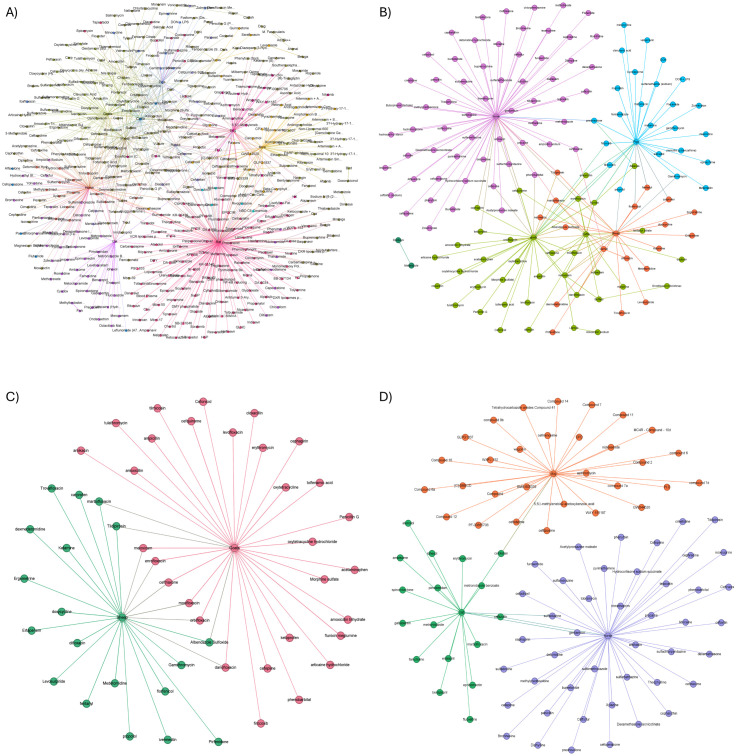
Primary imbalanced dataset showcasing (A) major clusters, (B) for the group ‘Ungulates’, (C) for the group ‘Small Ruminants’, (D) for the group ‘Companion Animals’, with clusters based on the drug administered per species. Clusters can be identified from its color.

Imbalanced Dataset: This prediction model was formulated using the raw/primary dataset for all CL_T_ values for all animal categories. Fig 6A represents a subgroup of clusters of the primary dataset based on species and drugs administered. The primary dataset was given as a candidate for the prediction models. ML models including tree-based models such as random forest (RF), and neural network models including multi-layer perceptron (MLP) were implemented here. Quantitative evaluations were considered (Table 3) and a cross-validation score (R^2^ Score ± Standard Deviation (STD)) of 0.791 ± 0.017 confirmed the actual efficiency of ML models in determining the CL_T_ data from the given PK study design parameters.

**Table 3:** Cross-validation scores for the hybrid ML CL_T_ dataset curated from the literature for all species.

ML Model	R^2^ Score ± STD			
	Raw Data	Undersampling	Oversampling	Simultaneous
RF	0.778 ± 0.020	0.775 ± 0.055	0.873 ± 0.003	0.872 ± 0.005
MLP	0.772 ± 0.021	0.783 ± 0.058	0.872 ± 0.004	0.871 ± 0.006
LR	0.725 ± 0.040	0.732 ± 0.063	0.829 ± 0.007	0.827 ± 0.004
RIDGE	0.727 ± 0.037	0.734 ± 0.063	0.829 ± 0.007	0.827 ± 0.004
LASSO	0.730 ± 0.034	0.738 ± 0.063	0.827 ± 0.007	0.824 ± 0.004
EN	0.657 ± 0.034	0.662 ± 0.042	0.750 ± 0.010	0.746 ± 0.009
k-NN	0.601 ± 0.020	0.611 ± 0.033	0.800 ± 0.014	0.793 ± 0.030
CART	0.791 ± 0.017	0.774 ± 0.051	0.873 ± 0.004	0.872 ± 0.005
SVM	0.691 ± 0.023	0.705 ± 0.050	0.837 ± 0.011	0.842 ± 0.006

Resampled Dataset: Balanced datasets generated using resampling techniques were also considered for the model implementation. Results shown in Table 3 revealed the importance of considering a balanced dataset for the CL_T_ prediction tasks. The same models including RF, and MLP with other regression models were considered for the imbalanced dataset as well. Compared to the imbalanced and undersampled models, both oversampled and simultaneously sampled sets generated improved cross-validation scores (Table 3). A cross-validation score greater than 0.871 ± 0.006 for the oversampled and simultaneously sampled sets reflects the efficiency of models especially CART, RF, and MLP, in handling continuous target data of the hybrid ML CL_T_ parameter.

### Case 2 (ungulates and all route forms – hybrid ML CL_T_)

Here the prediction models are considered a subset of the curated datasets focused on CL_T_ values for ungulates. Similar to the raw datasets (*Case 1*), this case also performs model efficiency validation with imbalanced and balanced datasets. The cross-validation scores (Table 4) show improved efficiency of the RF model (>85%) with imbalanced, LR model (~90%) with undersampled datasets, while RF, MLP, LR, RIDGE, and CART models (~92%) with oversampled and simultaneously sampled datasets. Fig 6B shows the clusters for the group ‘Ungulates’ based on species and drugs administered.

**Table 4:** Cross-validation scores for a subset of the hybrid ML CL_T_ dataset focusing on the group ungulates.

ML Model	R^2^ Score ± STD			
	Raw Data	Undersampling	Oversampling	Simultaneous
RF	0.876 ± 0.038	0.820 ± 0.069	0.939 ± 0.005	0.940 ± 0.011
MLP	0.797 ± 0.073	0.813 ± 0.051	0.939 ± 0.009	0.927 ± 0.018
LR	0.848 ± 0.122	0.898 ± 0.035	0.937 ± 0.014	0.934 ± 0.012
RIDGE	0.841 ± 0.038	0.836 ± 0.053	0.934 ± 0.009	0.932 ± 0.002
LASSO	0.774 ± 0.036	0.771 ± 0.080	0.825 ± 0.028	0.771 ± 0.037
EN	0.668 ± 0.017	0.630 ± 0.018	0.780 ± 0.022	0.776 ± 0.019
k-NN	0.811 ± 0.331	0.755 ± 0.029	0.927 ± 0.101	0.943 ± 0.008
CART	0.701 ± 0.203	0.787 ± 0.132	0.939 ± 0.005	0.939 ± 0.005
SVM	0.740 ± 0.025	0.637 ± 0.052	0.911 ± 0.028	0.906 ± 0.047

### Case 3 (small ruminants and all route forms – hybrid ML CL_T_)

This case considered the curated datasets focused on the species ‘Small ruminants’ (Fig 6C). Similar to the previous cases, all nine ML regression models were implemented, and their efficiencies were validated. In this dataset, LR outperformed the imbalanced and undersampled datasets (>87%) while MLP, RF, and CART models show the highest efficiency (~96%) with oversampled and simultaneously sampled datasets. Cross-validation scores obtained in this case are depicted in Table 5.

**Table 5:** Cross-validation scores for a subset of the hybrid ML CL_T_ dataset focusing on small ruminants.

ML Model	R^2^ Score ± STD			
	Raw Data	Undersampling	Oversampling	Simultaneous
RF	0.788 ± 0.140	0.703 ± 0.199	0.951 ± 0.012	0.960 ± 0.012
MLP	0.837 ± 0.094	0.778 ± 0.151	0.951 ± 0.010	0.957 ± 0.012
LR	0.870 ± 0.085	0.803 ± 0.094	0.945 ± 0.009	0.951 ± 0.008
RIDGE	0.837 ± 0.105	0.798 ± 0.120	0.941 ± 0.014	0.948 ± 0.008
LASSO	0.642 ± 0.175	0.608 ± 0.144	0.861 ± 0.072	0.848 ± 0.022
EN	0.593 ± 0.117	0.695 ± 0.072	0.850 ± 0.041	0.865 ± 0.019
k-NN	0.604 ± 0.013	0.845 ± 0.019	0.947 ± 0.021	0.936 ± 0.018
CART	0.723 ± 0.181	0.629 ± 0.287	0.952 ± 0.010	0.960 ± 0.012
SVM	0.781 ± 0.361	0.528 ± 0.147	0.903 ± 0.040	0.924 ± 0.016

### Case 4 (companion animals and all route forms – hybrid ML CL_T_)

This case considered the curated datasets focused on ‘Companion Animals’ (Fig 6D). Similar to the previous cases, all nine ML regression models were implemented and validated their efficiencies. In this case, the CART model outperformed all the resampling strategies (~91% and above), while MLP gave the highest score with imbalanced datasets (~79%). However, the tree-based models (CART, RF), MLP, and LR models had almost comparable outcomes. Cross-validation scores obtained in this case are depicted in Table 6.

**Table 6:** Cross-validation scores for a subset of the hybrid ML CL_T_ dataset focusing on companion animals.

ML Model	R^2^ Score ± STD			
	Raw Data	Undersampling	Oversampling	Simultaneous
RF	0.762 ± 0.074	0.868 ± 0.025	0.919 ± 0.015	0.919 ± 0.015
MLP	0.788 ± 0.150	0.835 ± 0.050	0.919 ± 0.014	0.919 ± 0.014
LR	0.777 ± 0.097	0.904 ± 0.026	0.920 ± 0.013	0.919 ± 0.015
RIDGE	0.742 ± 0.094	0.843 ± 0.017	0.913 ± 0.015	0.912 ± 0.015
LASSO	0.670 ± 0.052	0.677 ± 0.029	0.823 ± 0.030	0.834 ± 0.025
EN	0.591 ± 0.041	0.582 ± 0.011	0.669 ± 0.017	0.677 ± 0.013
k-NN	0.787 ± 0.108	0.770 ± 0.072	0.901 ± 0.009	0.906 ± 0.011
CART	0.769 ± 0.080	0.908 ± 0.024	0.921 ± 0.014	0.920 ± 0.015
SVM	0.692 ± 0.085	0.648 ± 0.023	0.794 ± 0.036	0.795 ± 0.025

### Case 5 (all route forms for different training:test data splitting – hybrid ML CL_T_)

Model validation was also performed by choosing different train:test dataset splitting criteria. It reveals a general sense of how the implemented model is performing on the new set. We applied this approach to all four scenarios (Case 1–4) previously discussed. Table 7 summarizes the performance metrics of selected ML models corresponding to various training:test splits selected for the study. RF was selected for the model validation phase based on the R^2^ scores achieved for the balanced datasets (Cases 1–4). For imbalanced and undersampled approaches, R^2^ score and EVS were unsurprisingly observed to decrease with an increase in the number of test samples, potentially caused by an insufficient amount of learning data corresponding to the test dataset. Other performance metrics such as MAE and RMSE also appear to be good despite the wide range of CL_T_ values in the dataset. Similarly, with an increase in the number of test datasets, we observe lower goodness of fit or R^2^ compared to the traditional 80:20 training:test data splitting, but with a minimum variation for oversampled and simultaneously sampled datasets.

**Table 7:** Performance Metrics R^2^, MAE, RMSE, and EVS scores for various data resampling methods for selected ML models.

Train:Test	Imbalanced				Undersampled				Oversampled				Simultaneous			
*Case 1: Raw dataset*																
	R^2^	MAE	RMSE	EVS	R^2^	MAE	RMSE	EVS	R^2^	MAE	RMSE	EVS	R^2^	MAE	RMSE	EVS
90:10	0.836	43.690	68.461	0.839	0.836	42.072	66.806	0.837	0.880	31.251	60.633	0.880	0.889	30.733	59.723	0.889
80:20	0.820	46.026	69.443	0.821	0.827	44.256	61.179	0.824	0.878	31.144	61.358	0.878	0.889	30.576	59.308	0.889
70:30	0.797	48.583	73.656	0.798	0.797	45.401	73.531	0.798	0.876	31.780	60.563	0.876	0.886	31.351	59.699	0.887
60:40	0.786	49.182	75.266	0.786	0.738	54.271	83.419	0.743	0.877	32.401	61.609	0.877	0.875	33.276	62.906	0.875
50:50	0.760	52.040	79.916	0.761	0.716	57.147	87.133	0.718	0.872	33.132	62.966	0.871	0.873	33.905	62.849	0.874
*Case 2: Ungulates*																
	R^2^	MAE	RMSE	EVS	R^2^	MAE	RMSE	EVS	R^2^	MAE	RMSE	EVS	R^2^	MAE	RMSE	EVS
90:10	0.932	2.059	3.243	0.935	0.924	2.649	4.688	0.926	0.966	1.835	4.276	0.967	0.975	2.11	3.694	0.971
80:20	0.904	3.661	6.566	0.912	0.915	3.756	5.640	0.917	0.954	2.306	4.821	0.954	0.949	2.662	4.985	0.950
70:30	0.895	4.205	6.916	0.898	0.895	4.252	6.374	0.897	0.952	2.261	4.701	0.952	0.956	2.431	4.675	0.956
60:40	0.885	4.749	6.471	0.889	0.848	4.622	7.362	0.852	0.949	2.573	5.064	0.949	0.954	2.728	4.866	0.954
50:50	0.858	5.562	7.681	0.858	0.824	4.900	7.940	0.829	0.945	2.713	5.283	0.946	0.944	3.063	5.341	0.944
*Case 3: Small Ruminants*																
	R^2^	MAE	RMSE	EVS	R^2^	MAE	RMSE	EVS	R^2^	MAE	RMSE	EVS	R^2^	MAE	RMSE	EVS
90:10	0.987	0.961	1.043	0.985	0.922	1.355	2.362	0.921	0.974	0.776	1.132	0.981	0.979	0.790	1.134	0.979
80:20	0.941	1.052	1.847	0.956	0.943	0.862	1.558	0.947	0.972	1.126	1.491	0.973	0.973	0.934	1.428	0.974
70:30	0.875	1.465	2.294	0.877	0.961	1.041	1.330	0.961	0.964	0.844	1.121	0.967	0.938	1.123	1.793	0.938
60:40	0.851	1.718	2.507	0.856	0.868	1.709	2.478	0.865	0.954	0.813	1.186	0.959	0.929	1.213	2.025	0.929
50:50	0.816	1.570	2.106	0.816	0.818	2.417	3.311	0.836	0.949	0.776	1.132	0.951	0.924	1.313	2.087	0.925
*Case 4: Companion Animals*																
	R^2^	MAE	RMSE	EVS	R^2^	MAE	RMSE	EVS	R^2^	MAE	RMSE	EVS	R^2^	MAE	RMSE	EVS
90:10	0.899	4.971	7.197	0.900	0.850	4.667	6.416	0.859	0.932	3.247	6.629	0.933	0.938	3.498	5.976	0.938
80:20	0.862	5.625	8.097	0.863	0.833	5.659	8.620	0.833	0.919	3.429	6.389	0.919	0.925	3.299	5.886	0.930
70:30	0.832	5.884	8.778	0.846	0.827	6.386	9.183	0.835	0.909	3.620	6.940	0.911	0.924	3.345	5.999	0.930
60:40	0.768	6.973	10.588	0.769	0.819	6.408	8.824	0.821	0.906	3.741	6.952	0.907	0.912	3.848	6.698	0.919
50:50	0.725	7.570	11.293	0.725	0.800	6.374	9.573	0.815	0.906	3.833	6.929	0.908	0.904	4.013	7.164	0.904

### Case 6 (IV only – true CL_T_)

The PK study design variables-based CL_T_ prediction model discussed in Case 1–5 did not take into account the influence of the bioavailability of a drug through various routes of administration [48,49]. As discussed earlier, the CL_T_ data we have curated from the literature includes both actual CL and apparent CL (CL/F). Therefore, the hybrid route independent ML CL_T_ that we have predicted so far is a combination of clearance values from both extravascular and intravascular drug administrations, thereby more closely representing CL/F. In **Case 6** cross-validation scores for various ML regression models were evaluated for datasets solely after intravenous (IV), IV Bolus, or Constant Rate Infusion, as the route of administration. Therefore, the CL_T_ dataset that we used in this case was restricted to actual/true CL values. The prediction results from the cross-validation process for the imbalanced and balanced dataset appeared promising. For the raw dataset, the highest accuracy score with a balanced dataset was around 0.865 ± 0.015, achieved by tree-based (RF, CART) and MLP models. With an imbalanced dataset, the highest scores were 0.787 ± 0.041 (MLP) and 0.781 ± 0.040 (RF). In the ungulate subset, tree-based and LR models showed improved performance, achieving scores of 0.936 ± 0.029 and 0.863 ± 0.023, respectively, outperforming slightly MLP (0.935 ± 0.016, 0.780 ± 0.073) for balanced and imbalanced datasets. For the small-ruminant subset, MLP achieved scores of 0.886 ± 0.082 (balanced) and 0.796 ± 0.140 (imbalanced), while tree-based and LR models scored 0.899 ± 0.084 (balanced) and 0.746 ± 0.187 (imbalanced). In the companion animal subset, tree-based and LR models outperformed MLP with a score of 0.961 ± 0.004 compared to 0.954 ± 0.010. However, with an imbalanced dataset, MLP (0.904 ± 0.027) performed better than RF (0.883 ± 0.030).

**Fig 7:**
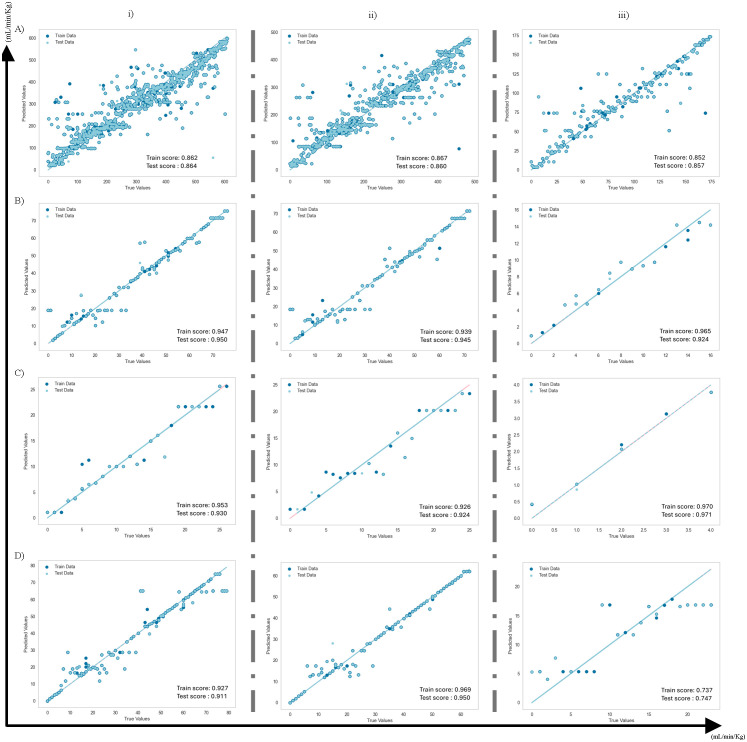
Goodness-of-fit metrics of RF model for the true vs predicted value for (A) all dataset, (B) ungulates dataset, (C) small ruminants, (D) companion animals dataset. True (Actual) values are fitted in the best-fit line (test data – cyan, train data – pink), and light, dark blue scatters correspond to the predicted values of test and training respectively. A vertical column separation (dashed line) is given for outcomes corresponding to three different groups: (i) datasets where all routes of administration are taken into consideration (hybrid ML CL); (ii) considering datasets with only route IV is selected, and (iii) datasets with all the routes except IV (non-IV) are considered. (70:30 train:test data splitting ratio).

After evaluating the imbalanced dataset using the cross-validation process, the ML models were independently adjusted by hyperparameter tuning (S2 File) to enhance prediction accuracy and explore different resampling techniques. Table 8 illustrates the prediction accuracy of the raw dataset (different animal categories), ungulates, small ruminants, and companion animals, for the RF models with 70:30 training:test data splitting for various resampling techniques. The low value of MAE and the prediction scores for the test dataset confirm that the model is effective even with an imbalanced dataset. Furthermore, Fig 7 (A–D) provides a comparative analysis of simultaneously sampled datasets (70:30 training:test data splitting ratio), highlighting the impact of considering the bioavailability of a drug through various routes of administration. The analysis shows results for three different groups separated with a dashed line: one where the dataset with all route forms is included (hybrid ML CL_T_), the second one where the dataset with only the intravenous (IV) route form is considered (true CL), and the third one where the dataset with IV route form is excluded (non-IV CL/F).

**Table 8:** Performance Metrics R^2^, MAE, RMSE, and EVS scores for various data resampling methods for Case 6 where Route = IV.

	Imbalanced				Undersampled			
Dataset	R^2^	MAE	RMSE	EVS	R^2^	MAE	RMSE	EVS
Raw dataset	0.781	17.536	24.365	0.781	0.781	14.668	24.401	0.781
Ungulates	0.901	4.609	5.881	0.902	0.892	4.176	5.570	0.894
Small Ruminants	0.877	1.887	2.674	0.880	0.907	1.573	2.372	0.925
Companion Animals	0.886	4.711	5.937	0.890	0.848	4.751	6.383	0.848
	**Oversampled**				**Simultaneous**			
Dataset	R^2^	MAE	RMSE	EVS	R^2^	MAE	RMSE	EVS
Raw dataset	0.852	11.120	19.560	0.852	0.864	10.930	17.291	0.884
Ungulates	0.951	2.511	4.816	0.951	0.952	2.762	4.660	0.952
Small Ruminants	0.932	1.5 92	2.056	0.936	0.940	1.304	1.903	0.941
Companion Animals	0.951	2.302	4.149	0.952	0.960	1.999	3.633	0.961

## Discussion and concluding remarks

Clearance is a critical PK parameter that is used to determine the rate of drug administration required to maintain steady-state concentrations. The present study focused on exploring the feasibility of predicting individual drug CL_T_ by considering drug and corresponding study design variables as the predictor variables. The drug CL_T_ prediction model implemented in this work relied solely on the literature data [24,27] and tested the possibility of predicting drug clearance, hybrid ML CL, true CL, and CL/F, by using individual drug, dose, route of administration, and species as independent or predictor variables in a machine learning approach. When compared to our previous MRL models [50], the CL_T_ dataset has some limitations such as the lack of multiple records for similar CL data and the fact that it is not a physiological true CL dataset, but rather a combination of both true CL and CL/F values. CL/F is influenced by both the drug’s clearance and its bioavailability, leading to a poor reflection of the actual clearance rate, especially in cases of low bioavailability. However, the model’s R^2^ score showed the efficiency of predicting CL_T_ from the imbalanced and balanced classes which is a positive note on the CL_T_ prediction. Similarly, other performance metrics obtained in the study also demonstrate the efficiency of ML models in accurately predicting the drug CL_T_ (hybrid ML CL, true CL) parameter from the selected features.

Compared to other classic PK and QSAR models [15,47,51,52], the CL_T_ data in this study is the hybrid ML CL_T_ because it includes both actual CL and apparent CL (CL/F) values curated from the scientific literature. This is a common attribute of clinical pharmacokinetic data and the values can be adjusted by bioavailability if known in a specific application. The ML models developed in this study predict PK hybrid ML CL_T_ (Case 1–5), and actual CL vs. CL/F (Case 6) parameters based on the drug, the animal subjects, route of administration, and dosage of the drug data available in the literature. In addition, this method does not use molecular, structural, physiological, or physiochemical descriptors of the drug/compound and concentrates on making predictions for individual drug included in the analyzed dataset and assesses the impact of its study design variables [50]. The models do not allow drug to drug extrapolations.

In the current study, different ML models including traditional SVC, k-NN, tree-based, and neural network-based models are implemented to validate the hybrid ML CL_T_, and true CL prediction accuracy. For the given dataset, tree-based models outperformed any other algorithms, especially for the resampling scenarios. Both qualitative and quantitative performance measures (Table 4) confirm the interpretation. The model’s performance potentially could be improved by training it with extensive, high-quality, and comprehensive datasets covering the majority of cases. This would enable the ML model to accurately estimate the clearance values of unknown data, even if that data is incomplete or has not been previously calculated. Like the model developed for MRL prediction [50], we can extend these studies to the prediction of any PK parameters provided we have enough data to train the model.

The performance measures depicted in the Tables and Figures are for the cleaned dataset generated after the outlier removal strategy. Here, a priori threshold criteria were applied to the training dataset in accordance with the recommendations from domain experts. In this way, the model addresses the anti-leakage concern so that it prevents the test data from influencing model fitting. Additionally, the influence of outlier removal on the model performance is determined by comparing the performance metrics with and without outlier removal. It enabled the model’s sensitivity assessment to extreme values. The efficacy and robustness of the proposed ML model for hybrid ML CL and true CL prediction were confirmed by the comparable R^2^ scores and RMSE scores in two scenarios.

The Food Animal Residue Avoidance and Depletion (FARAD) program, which started in 1982, assists producers, veterinarians, and allied professionals working with food-producing animals to avoid drug residues appearing in the food produced from these animals, avoiding residues is a PK problem. As part of the continued development of this computerized platform, we aim to generate diverse datasets and databases of PK parameters by performing automatic curation of scientific articles [27]. These databases serve as a knowledge resource summarizing the scientific literature related to PK studies and can support animal safety by limiting the need for in vivo PK procedures allowing rapid determination of strategies to mitigate drug and chemical residues in food animals under real-time clinical use scenarios. The present model is a useful tool to assess whether newly published CL data in a specific species is within expected values, and if not, to probe what conditions might explain the divergence when applied to a specific species for which drug residue predictions are required.

Data mining methodologies are applied to scientific articles or literature to extract data for research purposes. These methods treat the articles as a data resource, enabling researchers to gather and systematically analyze research papers, identify key concepts, relationships, and trends, and ultimately leverage the collective knowledge embedded within these resources [53–56]. We employed similar approaches in data collection, utilizing data extracted from HTML and PDF formats of scientific articles [57], whereas some recent studies relied on pre-existing databases such as openFDA [58], SRS [59], and BCI, Codex Alimentarius [50] as their primary data source. The data extraction and curation approaches used in this study are comparable to those presented in the studies of Gonzalez Hernandez, F. et al. [33] and Li, et al. [60], which also rely on scientific literature for pharmacokinetic datasets due to the absence of a robust database. In contrast, the studies presented in [21] focus on the manual curation of PK data and [61] focus on the OpenAI GPT-4 models for mining PK data from the scientific literature.

Some of the challenges that we faced in the current study are primarily associated with its data collection phase. Two pre-existing FARAD databases were utilized, and the initial approach involved manual curation of the existing data for this analysis [32], while the second technique involved the extraction of manuscript data from tables through an automated procedure [24,27]. The manual data extraction step was labor-intensive and time-consuming. In the automated table data extraction procedure, we had to deal with some specific scenarios including, but not limited to, (i) compounds or groups of compounds where the program was looking only for drug names based on the ATC drug classes [25,26], drug bank [62]; (ii) drug names such as *sultamicillin*, a prodrug for sulbactam and ampicillin was missing in our drug database [63]; and (iii) drug names mentioned as abbreviation specific to the curated manuscript [64]. However, the data collected was sufficient to create a foundation model that can be expanded upon to enhance a strategy of reinforcement learning in predicting drug CL_T_ [65]. The curated dataset had clearance values in different ranges and occurrences, resulting in an imbalanced dataset. The imbalanced distribution of classes is a key concern during the training phase of ML models as it can result in unsatisfactory models (classifiers/predictors), inaccurate or biased predictions as evident from the performance metrics of the raw dataset. The imbalanced class distributions were addressed by applying resampling methods such as oversampling, undersampling, and simultaneous sampling and proved the ML model’s potential to enhance the CL_T_, hybrid ML CL_T_, true CL, and CL/F prediction for a reliable dataset [42].

Table 9 presents a comprehensive analysis of various studies conducted on the estimation of drug clearance. Certain studies utilized both allometric and rule-based methods to estimate and predict drug clearance. These studies relied on statistical analyses to evaluate the model’s accuracy and performance [13,68,70,77,78]. Our proposed CL_T_ prediction model outperforms recent studies that have utilized ML models including artificial neural networks, and regression models, sacrificing prediction accuracy in the process [19,75,72]. An advantage of the proposed model is that it is not restricted to any limited set of drugs in the development phase of the model, but it is capable of accommodating combinations of the PK study design variables such as *drug-dosage-route_of_administration- animal* curated from the article repository [24,27]. This allows our model to be flexible enough to handle any new data records associated with CL prediction for *drug-dosage-route_of_administration-animal* input combination and could play a vital role in ensuring that veterinary animals receive safe, effective, and individualized drug therapy, especially in minor species (e.g., goats) where PK data is limited.

**Table 9:** A view of existing clearance-based prediction models. Included some of the studies based on the prediction method adopted.

Prediction Method	Knowledge Base	Discussions	Reference
Liver models – well-stirred, parallel tube, or dispersion	Hepatocyte or microsomal parameters	Usefulness of a well-stirred model and parallel tube model for in vivo and in vitro data prediction, respectively, has been identified.	Ito et al. 2004 [66]
Physiologic, empirical, drug-specific scaling factor (SF) methods + allometry	In vitro microsomal and/or preclinical animal data	Predicting in vivo intrinsic drug clearance and hepatic clearance are found to be more efficient with empirical SF.	Ito et al. 2005 [67]
Data-driven approach + Rule of Exponents (ROE)	CL data from rat, dog, or monkey models	Effectiveness of one- and two-species-based methods over three or more species in an allometrically based approach for predicting human drug clearance is discussed.	Tang et al. 2005 [68], 2007 [69]
ROE + fu Corrected Intercept Method (FCIM)	CL values of 40 drugs curated from the literature	Rational use of ROE and FCIM in human drug clearance prediction from animal data is found to be efficient for a wide variety of drugs.	Mahmood 2006 [70]
Simcyp® Population-Based ADME Simulator	Literature-based in vitro metabolism data and in vivo human clearance values	For the fifteen drugs that are selected for the study, median clearance values predicted fell within an acceptable 2-fold of observed values.	Howgate et al. 2006 [51]
Multiexponential allometry (MA) method	Preclinical data	Comparative estimation of human drug clearance using simple allometry (SA), monkey liver blood flow (MLBF), and MA methods led to a conclusion that the MA method has improved performance when used with preclinical data of more than 3 species.	Goteti et al. 2008 [71]
Artificial Neural Network (ANN)	In vitro intrinsic clearances (CL_int_) from published studies	Developed model seems to be useful in early drug discovery by predicting the PK parameter CL_int_ from human hepatocyte suspensions.	Paixão et al. 2010 [72]
Allometric Scaling	Drugs, Clearance, Volume of distribution at steady status, Body weight	Prediction of human CL and Vss: The study summarizes the feasibility of using log-log scale linear regression for predicting PK parameters across various species after intravenous administration.	Huang et al. 2015 [13]
Extended Clearance Classification System (ECCS)	Physicochemical properties, Passive membrane permeability	Predicting predominant clearance mechanism: As per the outcome, the proposed classification scheme was successful in early identification of the predominant clearance mechanism for the selected 92% of compounds.	Varma et al. 2015 [73]
PBPK Modeling	Preclinical ADME data	Usefulness of PBPK modeling in predicting human PK profiles from preclinical data has been established.	Zhuang et al. 2016 [74]
ML Regression models	Human intravenous PK data	Quantitative structure-activity relationship (QSAR) models developed for the prediction of PK parameters such as Vdss, CL, terminal half-life (t1/2), and fraction unbound in plasma (fu), outperformed the existing models, and opens up the possibility of developing in silico models to support early-stage drug discovery.	Wang et al. 2019 [75]
Random forest and XGBoost ML models	Human PK data with oral or intravenous route	Predicting compound PK parameters (target variable) using chemical structure information and dose as predictor variables	Miljković et al. 2021 [52]
PXB-mouse methods	Compounds, disposition, in vivo, in vitro data	Prediction of human CL of low-CL_int_ compounds showed better accuracy when compared to in vitro–in vivo extrapolation (IVIVE) and multispecies allometric (MA) scaling approaches.	Yoshida et al. 2022 [76]
Machine learning (ML) models	Chemical compounds, nonclinical data	Shows that the prediction of total body clearance and steady-state Vd (Vdss) using ML models that include missing-value imputation and feature selection can contribute significantly to efficient drug development.	Iwata et al. 2022 [19]
Classical extrapolative ML model, Augmented AS model	IV administered chemicals, across 15 mammalian species, laboratory animals, farm animals	Predicting total body CL of chemicals in farm animals using molecular descriptors and body weight.	Inauen et. Al 2025 [21]

In the early phases of the study (Cases 1–5), the ML models analyzed the target CL data independently from the absorption characteristics [48,49]. For drugs given through different routes of administration, their bioavailability is typically much lower than one, indicating incomplete absorption. By definition, IV administration has a bioavailability of 1. By considering the influence of various routes of administration on the bioavailability and the CL of a drug, the later phase of the research restricts the selection of datasets to route administration as *intravenous (IV), IV Bolus, or Constant Rate Infusion*. The regression scores proved satisfactory for the CL prediction model, despite the small dataset (Fig 7). This demonstrates the effectiveness of the model in predicting CL_T_ based on the study design variables such as drug, dose, and animal.

### Discussion of limitations and future directions

One of the limitations of our study is the small amount of data we used. However, the ML models were still good at predicting CL values. Because we curated the majority of the data automatically from scientific publications, the reported dose and CL values for a specific drug, route of administration, and animal, might vary across studies. This could affect model predictions. Since we used automated data extraction as one of the data extraction methods, the CL values came from both IV and non-IV routes of administration. The majority of the datasets compiled in this way come from IV studies, where CL is directly measured and remains unaffected by bioavailability. For the remaining data from extravascular routes, we used CL/F as it is the standard reported in the literature. This led us to choose hybrid ML CL models instead of total CL prediction models. Unlike other PK models that use molecular descriptors and physicochemical properties, our study is based on the PK study design variables for drugs included in the dataset. While high accuracy can be accomplished using drug, route of administration, dose, and animal as predictors, these factors alone lack mechanistic insight and cannot generalize across chemically diverse compounds (e.g., different drugs). Incorporating molecular descriptors could capture intrinsic structural and physicochemical properties affecting disposition, thereby potentially improving model trustworthiness and predictive reliability. To validate this aspect and progress our research, we are presently implementing advanced models that integrate ‘Chemical and Physical Properties: Computed Properties’ including Mw, LogP, TPSA from the PubChem database [79], along with the study design variables as predictor variables, resulting in an R^2^ score of ~ 0.82 for the combined imbalanced dataset in both cross-validation and 70:30 training:test data split scenarios; thereby showing the potential enhancement of the clearance prediction model.

The proposed CL prediction models, although preliminary due to the limited dataset, demonstrate the value of ML models in analyzing sparse PK parameter datasets. The models developed here will support future research efforts using automated techniques, including i) analyzing PK parameters, and ii) predicting PK parameters based on variables such as the drug itself, its molecular descriptors, physiochemical properties, dose, route of administration, and species. This work is part of the ongoing FARAD program, which requires real-time estimates of drug PK parameters to calculate evidence-based withdrawal intervals (WDIs) for specific drugs in specific species. CL quantifies the rate at which a drug is eliminated from an animal’s system, directly influencing the duration of WDIs. A higher CL rate typically leads to a shorter withdrawal period, while a lower clearance rate extends the time required for drug residues to fall below regulatory limits [80]. These intervals are essential to ensure that food animal products are free from potentially harmful drug residues following extra-label drug use. Our data-mining project aims to build a comprehensive pharmaceutical database that will enhance FARAD’s capacity to provide timely and accurate residue avoidance recommendations. This objective is particularly critical given the increasing global trade of minor species across regulatory boundaries, lack of extensive PK data in minor species, as well as the growing restrictions on conducting live animal studies to generate toxicological endpoints. Leveraging ML models to predict PK parameters may assist in addressing these challenges and contributing to improved food safety and public health outcomes.

In summary, this paper introduces an automated method for data mining that can effectively estimate CL_T_ for drugs in a new dataset (PK study design variables test data), and this method can also be extended to estimate drug CL that is not reported or is currently missing. Databases, such as those curated in this study, play a vital role in the development of ML algorithms that effectively estimate PK parameters, such as CL. Overall, the models in this study were efficient at accurately estimating and predicting CL_T_ values, achieving very low MAE and RMSE scores, as well as achieving an EVS and R^2^ score closer to 1, which is indicative of high model efficiency. The models we employed to estimate CL_T_ for a subset of groups such as ungulates and small ruminants had a higher degree of predictive accuracy, which might be due to a limited range of CL_T_ values and narrow species selection. Although the results demonstrate that study design variables can facilitate the prediction of drug CL, the approach is constrained by its focus on existing drugs and the exclusion of comprehensive drug development factors. Moreover, while the cross-species extrapolation tool shows potential, its effectiveness is constrained by the practical difficulties of obtaining accurate CL values across different species and study contexts. Future research should therefore enhance the model to incorporate additional biologically relevant predictors such as molecular descriptors and assess the performance of the proposed cross-species extrapolation tool in early-stage compound evaluation, where empirical CL data is often scarce [81,82]. Because there was a wide range of CL_T_ values, our study has limitations when it comes to using the leave-one-out cross-validation technique for various species or drug groups. Additionally, the current dataset considered in the model lacks consistent metadata, such as study or publication year and laboratory origin. However, it is worth mentioning that incorporating these validation schemes in future versions of the model may provide additional insight into its robustness and broader applicability to PK parameter prediction as well as model generalizability to entirely unseen dataset and model sensitivity to evolving experimental practices and source-specific reporting patterns.

In general, machine-learning models implemented in this study demonstrated robust and reliable clearance prediction using curated PK-literature data, despite the challenges including inherent sparsity, noise, and class imbalance. Feature importance and SHAP analyses showed that predictions were primarily influenced by drug related properties, dosage, species, and route of administration. This aligns with the established PK principles and supporting interpretability for medicinal chemists and DMPK scientists. The model performance was stable across several data-splitting approaches and aligned well with observed outcomes, with no systematic contradictions to known PK behavior. Though direct benchmarking against traditional allometry or IVIVE approaches was not conducted, performance was comparable to ranges reported in the literature. Future work will focus on the benchmarking with mechanistic methods, and model development incorporating expanded molecular descriptors and high-quality human data to improve translational relevance and support a comprehensive One Health predictive framework.

## Supporting information

S1 File Machine Learning-Based Unified Models for Predicting Drug Clearance from Pharmacokinetic Animal and Study Design Variables. (HTML)

S2 File Best parameters identified for different data sampling methods to fit the 9 ML regression models. Random Forest (RF), Multi-Layer Perceptron (MLP), Linear Regression (LR), Ridge Regression (RIDGE), Lasso Regression (LASSO), Elastic Net (EN), K-Neighbors (k-NN), Classification and Regression Trees (CART), and Support Vector Regressor (SVR) for six different cases. Case 1: all animal categories and route forms. Case 2: ungulates and all route forms, Case 3: small ruminants and all route forms, Case 4: companion animals and all route forms for hybrid ML CLT prediction, and Case 6: IV only dataset, CLT prediction [6A: all animal categories, 6B: ungulates, 6C: small ruminants, 6D: companion animals].
(PDF)
