# Supplementary material for: Machine learning-based unified models for predicting drug clearance from pharmacokinetic animal and study design variables
Source: PLoS One. 2026 May 6;21(5):e0346432. doi: 10.1371/journal.pone.0346432 (PMC13148688; doi:10.1371/journal.pone.0346432)
Supplement: S1 File — (HTML) [file pone.0346432.s001.html]

### Supplementary Material S1

### Machine Learning-Based Unified Models for Predicting Drug Clearance from Pharmacokinetic Animal and Study Design Variables

Remya Ampadi Ramachandran1,2,3, Lisa A. Tell4, Melissa Mercer5, Xuan Xu1,2, Nuwan Millagaha Gedara1,6, Maaike Ottoline Clapham4, Zhoumeng Lin7,8, Jim E. Riviere1,2,9, Majid Jaberi-Douraki1,2,3,\*

11DATA Consortium, www.1DATA.life, Kansas State University Olathe, Olathe, KS, USA;
2Food Animal Residue Avoidance and Databank Program (FARAD), Kansas State University Olathe, Olathe, KS, USA;
3Department of Mathematics, Kansas State University, Manhattan, KS, USA;
4FARAD, Department of Medicine and Epidemiology, School of Veterinary Medicine, University of California-Davis, Davis, CA, USA;
5Department of Biomedical Sciences and Pathobiology, VA-MD College of Veterinary Medicine, Virginia Tech, VA, USA;
6College of Business, Loyola University, New Orleans, LA, USA;
7Department of Environmental and Global Health, College of Public Health and Health Professions, University of Florida, Gainesville, FL, USA;
8Center for Environmental and Human Toxicology, University of Florida, Gainesville, FL, USA;
9FARAD, Department of Population Health and Pathobiology, College of Veterinary Medicine, North Carolina State University, Raleigh, NC, USA.

\*Corresponding Author: jaberi@k-state.edu

#### Legend

Amphibians

Avian/Birds

Canidae

Crustaceans

Elephantidae

Equids

Felidae

Fish

Lagomorphs

Marsupials

Mustelids

Pinnipeds

Primates

Psuedoruminants

Reptiles

Rodents

Ruminants

Small Ruminants

Suidae


# 


# 


0%
