# Supplementary material for: Machine learning-based unified models for predicting drug clearance from pharmacokinetic animal and study design variables
Source: PLoS One. 2026 May 6;21(5):e0346432. doi: 10.1371/journal.pone.0346432 (PMC13148688; doi:10.1371/journal.pone.0346432)
Supplement: S2 File — Random Forest (RF), Multi-Layer Perceptron (MLP), Linear Regression (LR), Ridge Regression (RIDGE), Lasso Regression (LASSO), Elastic Net (EN), K-Neighbors (k-NN), Classification and Regression Trees (CART), and Support Vector Regressor (SVR) for six different cases. Case 1: all animal categories and route forms. Case 2: ungulates and all route forms, Case 3: small ruminants and all route forms, Case 4: companion animals and all route forms for hybrid ML CLT prediction, and Case 6: IV only dataset, CLT prediction [6A: all animal categories, 6B: ungulates, 6C: small ruminants, 6D: companion animals]. (PDF) [file pone.0346432.s002.pdf]

Supplementary Material S2

Machine Learning-Based Unified Models for Predicting Drug Clearance from Pharmacokinetic Animal and Study Design Variables

Remya Ampadi Ramachandran[1,2,3], Lisa A. Tell[4], Melissa Mercer[5], Xuan Xu[1,2], Nuwan Millagaha Gedara[1,6], Maaike Ottoline Clapham[4], Zhoumeng Lin[7,8], Jim E. Riviere[1,2,9], Majid Jaberi-Douraki[1,2,3,*]

[1] 1DATA Consortium, www.1DATA.life, Kansas State University Olathe, Olathe, KS, USA.

[2] Food Animal Residue Avoidance and Databank Program (FARAD), Kansas State University Olathe, Olathe, KS, USA.

[3] Department of Mathematics, Kansas State University, Manhattan, KS, United States

[4] FARAD, Department of Medicine and Epidemiology, School of Veterinary Medicine, University of California-Davis, Davis, CA, USA.

[5] Department of Biomedical Sciences and Pathobiology, VA-MD College of Veterinary Medicine, Virginia Tech, VA, USA.

[6] College of Business, Loyola University, New Orleans, LA, USA.

[7] Department of Environmental and Global Health, College of Public Health and Health Professions, University of Florida, Gainesville, FL, USA.

[8] Center for Environmental and Human Toxicology, University of Florida, Gainesville, FL, USA.

[9] FARAD, Department of Population Health and Pathobiology, College of Veterinary Medicine, North Carolina State University, Raleigh, NC, USA.

* Corresponding Author: jaberi@k-state.edu

Table S2. Best parameters identified for different data sampling methods to fit the 9 ML regression models: Random Forest (RF), Multi-Layer Perceptron (MLP), Linear Regression (LR), Ridge Regression (RIDGE), Lasso Regression (LASSO), Elastic Net (EN), K-Neighbors (k-NN), Classification and Regression Trees (CART), and Support Vector Regressor (SVR) for six different cases. Case 1: all animal categories and route forms. Case 2: ungulates and all route forms, Case 3: small ruminants and all route forms, Case 4: companion animals and all route forms for hybrid ML CL_T prediction, and Case 6: IV only dataset, CL_T prediction [6A: all animal categories, 6B: ungulates, 6C: small ruminants, 6D: companion animals].

Case	ML Model	Best estimators for different re-sampling strategies			
		Imbalanced	Undersampling	Oversampling	Simultaneous
1	RF	'bootstrap': True, 'max_depth': 5, 'min_samples_leaf': 1, 'n_estimators': 50	'bootstrap': True, 'max_depth': 50, 'min_samples_leaf': 1, 'n_estimators': 100	'bootstrap': True, 'max_depth': 15, 'min_samples_leaf': 1, 'n_estimators': 150	'bootstrap': True, 'max_depth': 15, 'min_samples_leaf': 1, 'n_estimators': 100
	MLP	'activation': 'relu', 'alpha': 0.0001, 'hidden_layer_sizes': (20,), 'learning_rate': 'adaptive', 'solver': 'adam'	'activation': 'relu', 'alpha': 0.0001, 'hidden_layer_sizes': (20,), 'learning_rate': 'constant', 'solver': 'adam'	'activation': 'relu', 'alpha': 0.0001, 'hidden_layer_sizes': (20,), 'learning_rate': 'constant', 'solver': 'adam'	'activation': 'relu', 'alpha': 0.0001, 'hidden_layer_sizes': (20,), 'learning_rate': 'constant', 'solver': 'adam'
	LR	'positive': False, 'n_jobs': 1, 'fit_intercept': False, 'copy_X': True	'positive': False, 'n_jobs': 1, 'fit_intercept': True, 'copy_X': True	'positive': False, 'n_jobs': 1, 'fit_intercept': True, 'copy_X': True	'positive': False, 'n_jobs': 1, 'fit_intercept': True, 'copy_X': True
	RIDGE	'alpha': 0.1	'alpha': 0.1	'alpha': 0.1	'alpha': 0.1
	LASSO	'alpha': 0.1	'alpha': 0.1	'alpha': 0.1	'alpha': 0.1
	EN	'alpha': 0.1, 'l1_ratio': 0.8	'alpha': 0.1, 'l1_ratio': 0.8	'alpha': 0.1, 'l1_ratio': 0.8	'alpha': 0.1, 'l1_ratio': 0.8
	k-NN	'metric': 'euclidean', 'n_neighbors': 3, 'weights': 'distance'	'metric': 'euclidean', 'n_neighbors': 11, 'weights': 'distance'	'metric': 'euclidean', 'n_neighbors': 11, 'weights': 'distance'	'metric': 'euclidean', 'n_neighbors': 11, 'weights': 'distance'
	CART	'max_depth': 25, 'min_samples_leaf': 1,	'max_depth': 25, 'min_samples_leaf': 1,	'max_depth': 25, 'min_samples_leaf': 1,	'max_depth': 25, 'min_samples_leaf': 1,

		'min_samples_split': 2	'min_samples_split': 5	'min_samples_split': 5	'min_samples_split': 2
	SVR	'C': 100, 'gamma': 1, 'kernel': 'rbf'	'C': 100, 'gamma': 1, 'kernel': 'rbf'	'C': 100, 'gamma': 'scale', 'kernel': 'rbf'	'C': 100, 'gamma': 1, 'kernel': 'rbf'
2	RF	'bootstrap': False, 'max_depth': 15, 'min_samples_leaf': 1, 'n_estimators': 100	'bootstrap': False, 'max_depth': 15, 'min_samples_leaf': 1, 'n_estimators': 50	'bootstrap': True, 'max_depth': 15, 'min_samples_leaf': 1, 'n_estimators': 50	'bootstrap': True, 'max_depth': 15, 'min_samples_leaf': 1, 'n_estimators': 50
	MLP	'activation': 'tanh', 'alpha': 0.0001, 'hidden_layer_sizes': (20,), 'learning_rate': 'adaptive', 'solver': 'adam'	'activation': 'tanh', 'alpha': 0.0001, 'hidden_layer_sizes': (20,), 'learning_rate': 'adaptive', 'solver': 'adam'	'activation': 'tanh', 'alpha': 0.0001, 'hidden_layer_sizes': (20,), 'learning_rate': 'adaptive', 'solver': 'adam'	'activation': 'tanh', 'alpha': 0.05, 'hidden_layer_sizes': (20,), 'learning_rate': 'constant', 'solver': 'adam'
	LR	'copy_X': True, 'fit_intercept': True, 'n_jobs': 1, 'positive': False	'copy_X': True, 'fit_intercept': True, 'n_jobs': 1, 'positive': False	'copy_X': True, 'fit_intercept': True, 'n_jobs': 1, 'positive': False	'copy_X': True, 'fit_intercept': False, 'n_jobs': 1, 'positive': False
	RIDGE	'alpha': 0.1	'alpha': 0.1	'alpha': 0.1	'alpha': 0.1
	LASSO	'alpha': 0.1	'alpha': 0.1	'alpha': 0.1	'alpha': 0.1
	EN	'alpha': 0.1, 'l1_ratio': 0.8	'alpha': 0.1, 'l1_ratio': 0.8	'alpha': 0.1, 'l1_ratio': 0.8	'alpha': 0.1, 'l1_ratio': 0.8
	k-NN	'metric': 'euclidean', 'n_neighbors': 5, 'weights': 'distance'	'metric': 'euclidean', 'n_neighbors': 9, 'weights': 'distance'	'metric': 'euclidean', 'n_neighbors': 11, 'weights': 'distance'	'metric': 'euclidean', 'n_neighbors': 9, 'weights': 'distance'
	CART	'max_depth': 15, 'min_samples_leaf': 1, 'min_samples_split': 2	'max_depth': 25, 'min_samples_leaf': 1, 'min_samples_split': 2	'max_depth': 25, 'min_samples_leaf': 1, 'min_samples_split': 2	'max_depth': 25, 'min_samples_leaf': 1, 'min_samples_split': 10

	SVR	'C': 100, 'gamma': 0.1, 'kernel': 'rbf'	'C': 100, 'gamma': 'scale', 'kernel': 'linear'	'C': 100, 'gamma': 0.1, 'kernel': 'rbf'	'C': 100, 'gamma': 'scale', 'kernel': 'rbf'
3	RF	'bootstrap': False, 'max_depth': 5, 'min_samples_leaf': 1, 'n_estimators': 150	'bootstrap': True, 'max_depth': 5, 'min_samples_leaf': 1, 'n_estimators': 50	'bootstrap': False, 'max_depth': 15, 'min_samples_leaf': 1, 'n_estimators': 50	'bootstrap': True, 'max_depth': 10, 'min_samples_leaf': 1, 'n_estimators': 200
	MLP	'activation': 'tanh', 'alpha': 0.0001, 'hidden_layer_sizes': (10, 30, 10), 'learning_rate': 'constant', 'solver': 'adam'	'activation': 'tanh', 'alpha': 0.05, 'hidden_layer_sizes': (10, 30, 10), 'learning_rate': 'constant', 'solver': 'adam'	'activation': 'tanh', 'alpha': 0.0001, 'hidden_layer_sizes': (20,), 'learning_rate': 'constant', 'solver': 'adam'	'activation': 'tanh', 'alpha': 0.0001, 'hidden_layer_sizes': (20,), 'learning_rate': 'adaptive', 'solver': 'adam'
	LR	'copy_X': True, 'fit_intercept': True, 'n_jobs': 1, 'positive': False	'copy_X': True, 'fit_intercept': True, 'n_jobs': 1, 'positive': False	'copy_X': True, 'fit_intercept': True, 'n_jobs': 1, 'positive': False	'copy_X': True, 'fit_intercept': False, 'n_jobs': 1, 'positive': False
	RIDGE	'alpha': 0.1	'alpha': 0.1	'alpha': 0.1	'alpha': 0.1
	LASSO	'alpha': 0.1	'alpha': 0.1	'alpha': 0.1	'alpha': 0.1
	EN	'alpha': 0.1, 'l1_ratio': 0.8	'alpha': 0.1, 'l1_ratio': 0.8	'alpha': 0.1, 'l1_ratio': 0.8	'alpha': 0.1, 'l1_ratio': 0.8
	k-NN	'metric': 'manhattan', 'n_neighbors': 5, 'weights': 'distance'	'metric': 'euclidean', 'n_neighbors': 7, 'weights': 'distance'	'metric': 'manhattan', 'n_neighbors': 5, 'weights': 'distance'	'metric': 'euclidean', 'n_neighbors': 3, 'weights': 'distance'
	CART	'max_depth': 4, 'min_samples_leaf': 2, 'min_samples_split': 2	'max_depth': 5, 'min_samples_leaf': 2, 'min_samples_split': 2	'max_depth': 25, 'min_samples_leaf': 1, 'min_samples_split': 2	'max_depth': 25, 'min_samples_leaf': 1, 'min_samples_split': 10
	SVR	'C': 10, 'gamma': 'scale', 'kernel': 'linear'	'C': 100, 'gamma': 1, 'kernel': 'rbf'	'C': 100, 'gamma': 'scale', 'kernel': 'rbf'	'C': 10, 'gamma': 'scale', 'kernel': 'rbf'

4	RF	'bootstrap': True, 'max_depth': 15, 'min_samples_leaf': 1, 'n_estimators': 150	'bootstrap': True, 'max_depth': 15, 'min_samples_leaf': 1, 'n_estimators': 50	'bootstrap': True, 'max_depth': 15, 'min_samples_leaf': 1, 'n_estimators': 150	'bootstrap': True, 'max_depth': 15, 'min_samples_leaf': 1, 'n_estimators': 50
	MLP	'activation': 'tanh', 'alpha': 0.0001, 'hidden_layer_sizes': (20,), 'learning_rate': 'constant', 'solver': 'adam'	'activation': 'tanh', 'alpha': 0.0001, 'hidden_layer_sizes': (20,), 'learning_rate': 'constant', 'solver': 'adam'	'activation': 'tanh', 'alpha': 0.0001, 'hidden_layer_sizes': (20,), 'learning_rate': 'constant', 'solver': 'adam'	'activation': 'tanh', 'alpha': 0.0001, 'hidden_layer_sizes': (20,), 'learning_rate': 'adaptive', 'solver': 'adam'
	LR	'copy_X': True, 'fit_intercept': False, 'n_jobs': 1, 'positive': False	'copy_X': True, 'fit_intercept': True, 'n_jobs': 1, 'positive': False	'copy_X': True, 'fit_intercept': False, 'n_jobs': 1, 'positive': False	'copy_X': True, 'fit_intercept': False, 'n_jobs': 1, 'positive': False
	RIDGE	'alpha': 0.1	'alpha': 0.1	'alpha': 0.1	'alpha': 0.1
	LASSO	'alpha': 0.1	'alpha': 0.1	'alpha': 0.1	'alpha': 0.1
	EN	'alpha': 0.1, 'l1_ratio': 0.8	'alpha': 0.1, 'l1_ratio': 0.8	'alpha': 0.1, 'l1_ratio': 0.8	'alpha': 0.1, 'l1_ratio': 0.8
	k-NN	'metric': 'manhattan', 'n_neighbors': 5, 'weights': 'distance'	'metric': 'manhattan', 'n_neighbors': 5, 'weights': 'distance'	'metric': 'euclidean', 'n_neighbors': 11, 'weights': 'distance'	'metric': 'manhattan', 'n_neighbors': 11, 'weights': 'distance'
	CART	'max_depth': 25, 'min_samples_leaf': 1, 'min_samples_split': 2	'max_depth': 25, 'min_samples_leaf': 1, 'min_samples_split': 2	'max_depth': 25, 'min_samples_leaf': 1, 'min_samples_split': 5	'max_depth': 25, 'min_samples_leaf': 2, 'min_samples_split': 10
	SVR	'C': 100, 'gamma': 'scale', 'kernel': 'linear'	'C': 100, 'gamma': 'scale', 'kernel': 'linear'	'C': 10, 'gamma': 'scale', 'kernel': 'linear'	'C': 100, 'gamma': 'scale', 'kernel': 'linear'
6A	RF	'bootstrap': True, 'max_depth': 15, 'min_samples_leaf':	'bootstrap': True, 'max_depth': 15, 'min_samples_leaf':	'bootstrap': True, 'max_depth': 15, 'min_samples_leaf':	'bootstrap': True, 'max_depth': 15, 'min_samples_leaf':

		1, 'n_estimators': 150	1, 'n_estimators': 100	1, 'n_estimators': 150	1, 'n_estimators': 200
	MLP	'activation': 'tanh', 'alpha': 0.05, 'hidden_layer_sizes': (20,), 'learning_rate': 'constant', 'solver': 'adam'	'activation': 'relu', 'alpha': 0.0001, 'hidden_layer_sizes': (20,), 'learning_rate': 'adaptive', 'solver': 'adam'	'activation': 'relu', 'alpha': 0.05, 'hidden_layer_sizes': (20,), 'learning_rate': 'adaptive', 'solver': 'adam'	'activation': 'relu', 'alpha': 0.0001, 'hidden_layer_sizes': (20,), 'learning_rate': 'constant', 'solver': 'adam'
6B	RF	'bootstrap': True, 'max_depth': 5, 'min_samples_leaf': 1, 'n_estimators': 100	'bootstrap': False, 'max_depth': 15, 'min_samples_leaf': 1, 'n_estimators': 50	'bootstrap': False, 'max_depth': 15, 'min_samples_leaf': 1, 'n_estimators': 50	'bootstrap': False, 'max_depth': 15, 'min_samples_leaf': 1, 'n_estimators': 150
	MLP	'activation': 'tanh', 'alpha': 0.0001, 'hidden_layer_sizes': (20,), 'learning_rate': 'adaptive', 'solver': 'adam'	'activation': 'tanh', 'alpha': 0.0001, 'hidden_layer_sizes': (20,), 'learning_rate': 'constant', 'solver': 'adam'	'activation': 'tanh', 'alpha': 0.0001, 'hidden_layer_sizes': (20,), 'learning_rate': 'constant', 'solver': 'adam'	'activation': 'tanh', 'alpha': 0.05, 'hidden_layer_sizes': (20,), 'learning_rate': 'constant', 'solver': 'adam'
6C	RF	'bootstrap': False, 'max_depth': 5, 'min_samples_leaf': 1, 'n_estimators': 50	bootstrap': True, 'max_depth': 5, 'min_samples_leaf': 1, 'n_estimators': 50	'bootstrap': False, 'max_depth': 5, 'min_samples_leaf': 1, 'n_estimators': 50	'bootstrap': False, 'max_depth': 5, 'min_samples_leaf': 1, 'n_estimators': 50
	MLP	'activation': 'tanh', 'alpha': 0.0001, 'hidden_layer_sizes': (10, 30, 10), 'learning_rate': 'constant', 'solver': 'adam'	'activation': 'tanh', 'alpha': 0.0001, 'hidden_layer_sizes': (10, 30, 10), 'learning_rate': 'adaptive', 'solver': 'adam'	'activation': 'tanh', 'alpha': 0.0001, 'hidden_layer_sizes': (10, 30, 10), 'learning_rate': 'adaptive', 'solver': 'adam'	'activation': 'tanh', 'alpha': 0.05, 'hidden_layer_sizes': (20,), 'learning_rate': 'adaptive', 'solver': 'adam'
6D	RF	'bootstrap': False, 'max_depth': 10, 'min_samples_leaf':	'bootstrap': False, 'max_depth': 10, 'min_samples_leaf':	'bootstrap': True, 'max_depth': 10, 'min_samples_leaf':	'bootstrap': True, 'max_depth': 15, 'min_samples_leaf':

		1, 'n_estimators': 100	2, 'n_estimators': 100	1, 'n_estimators': 150	1, 'n_estimators': 100
	MLP	'activation': 'tanh', 'alpha': 0.0001, 'hidden_layer_sizes': (20,), 'learning_rate': 'adaptive', 'solver': 'adam'	'activation': 'tanh', 'alpha': 0.0001, 'hidden_layer_sizes': (20,), 'learning_rate': 'constant', 'solver': 'adam'	'activation': 'tanh', 'alpha': 0.0001, 'hidden_layer_sizes': (20,), 'learning_rate': 'constant', 'solver': 'adam'	'activation': 'tanh', 'alpha': 0.05, 'hidden_layer_sizes': (20,), 'learning_rate': 'adaptive', 'solver': 'adam

Note: Estimators selected for the ML models

1. Random Forest (RF)

bootstrap: whether to use bootstrapping when creating trees
n_estimators: number of trees in the forest
max_depth: maximum depth of each individual tree
min_samples_leaf: minimum number of samples required at a leaf node

2. Multi-Layer Perceptron (MLP)

activation: specifies the non-linear activation function applied to each neuron in the hidden layers
alpha: represents the regularization parameter
hidden_layer_sizes: defines the structure of the hidden layers by specifying the number of neurons in each layer
learning_rate: controls the step size taken during the weight updates during training
solver: the optimization algorithm used to update the model's weights (adam, lbfgs, stochastic gradient descent (sgd))

3. Linear Regression (LR)

positive: keeps the model coefficients to be positive
n_jobs: controls how many CPU cores to use during model training
fit_intercept: boolean parameter that determines whether the model should include an intercept term in the regression equation
copy_X: controls whether the input feature matrix is copied before being modified during model fitting

4. Ridge Regression (RIDGE)

alpha: controls the overall strength of regularization

5. Lasso Regression (LASSO)

alpha: controls the overall strength of regularization

6. Elastic Net (EN)<br>alpha: controls the overall strength of regularization<br>l1_ratio: controls the penalties (L1 penalty and L2 penalty) that we impose on the model
7. K-Neighbors (k-NN)<br>metric: distance metric to calculate how far apart two data points are (euclidean, manhattan, cosine similarity)<br>n_neighbors: (k) : sets the number of nearest neighbors for making the prediction<br>weights: determines how much each neighbor contributes to the prediction
8. Classification and Regression Trees (CART)<br>max_depth: maximum number of levels a decision tree can have<br>min_samples_leaf: minimum number of data points required in each leaf node<br>min_samples_split: minimum number of data points needed to split an internal node
9. Support Vector Regressor (SVR)<br>C: regularization parameter that controls the penalty for misclassified data points<br>gamma: kernel coefficient that controls decision boundary<br>kernel: determines how data points are compared to identify similarities (linear, polynomial, radial basis function (rbf))
